# Supplementary figures and images for: Are Roma People Descended from the Punjab Region of Pakistan: A Y-Chromosomal Perspective
Source: Genes (Basel). 2022 Mar 17;13(3):532. doi: 10.3390/genes13030532 (PMC8951058; doi:10.3390/genes13030532)

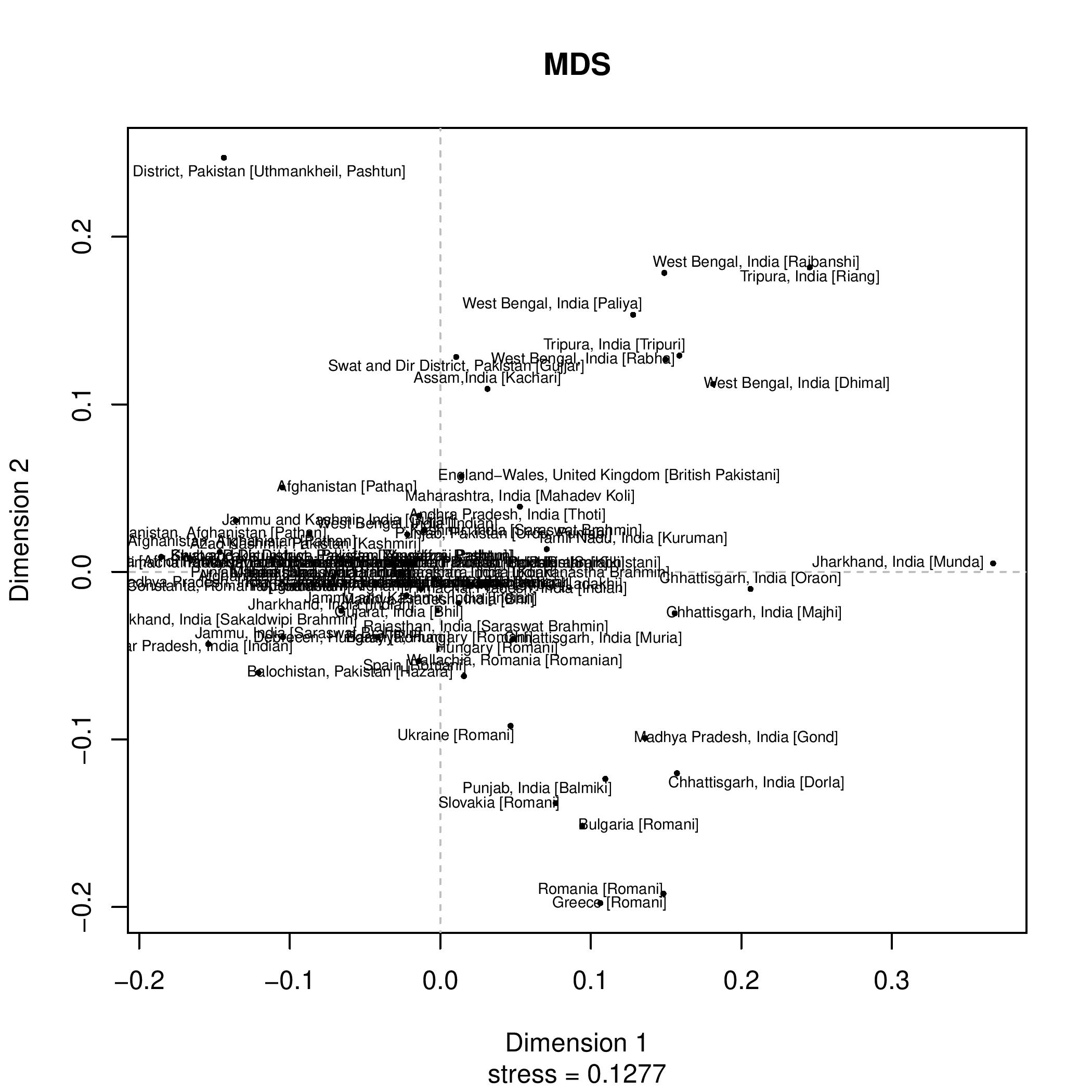

Supplement: Supplementary file 1 [file genes-13-00532-s001.zip › Supplementary Figure S1. MDS Plot Rst_81.jpg]

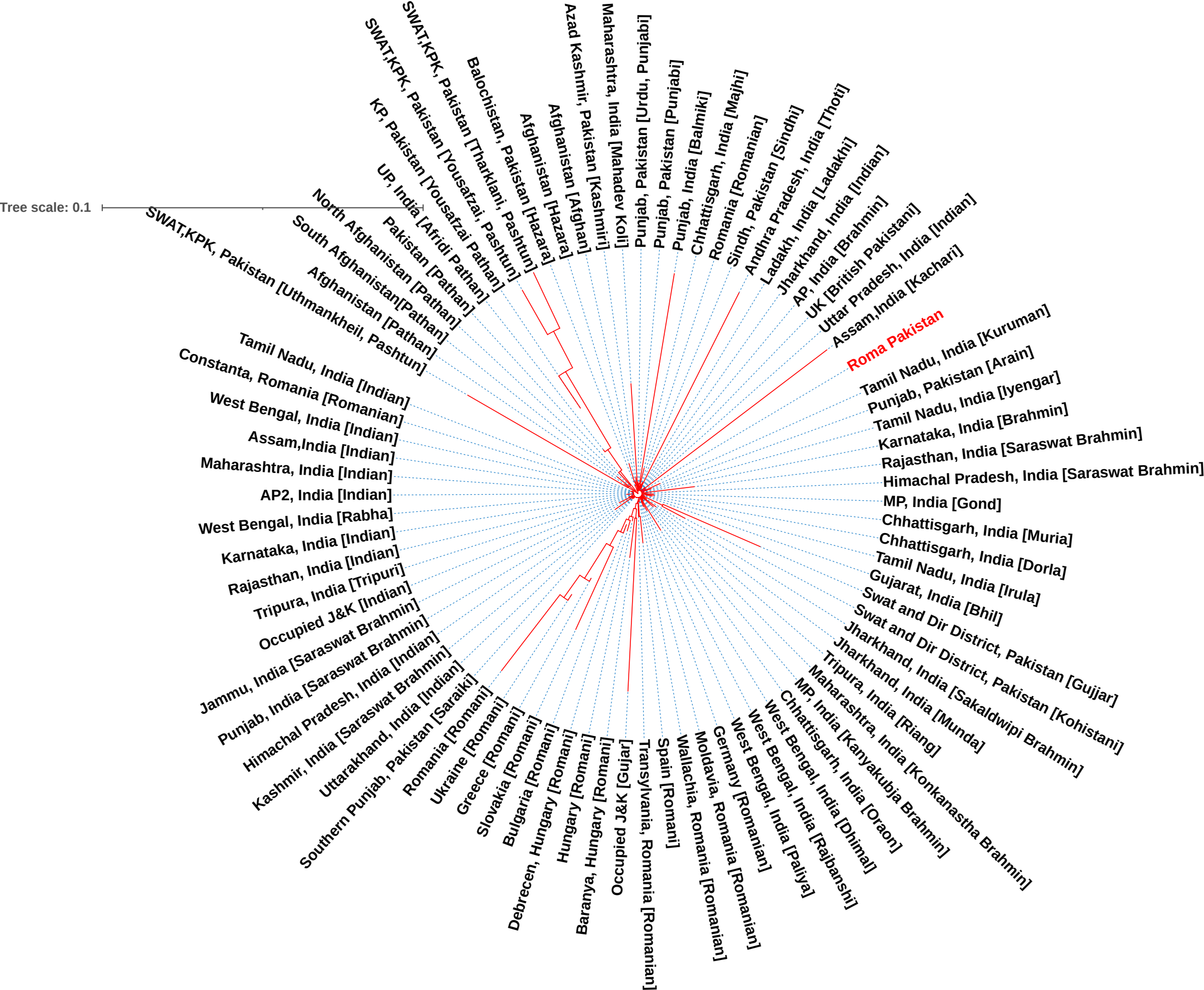

Supplement: Supplementary file 1 [file genes-13-00532-s001.zip › Supplementary Figure S2. Neighbor-joining phylogenetic tree for 81 populations from Pakistan, India, Afghanistan and Europe based on a distance matrix of Fst.pdf]

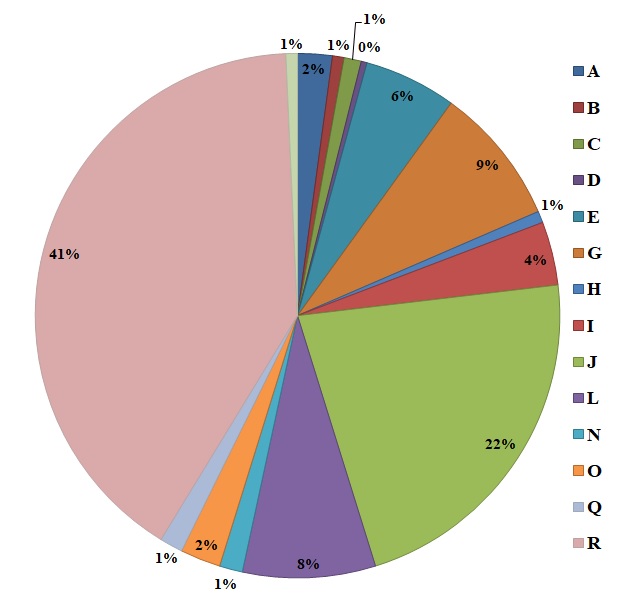

Supplement: Supplementary file 1 [file genes-13-00532-s001.zip › Supplementary Figure S3. Haploroups in Roma PK.jpg]

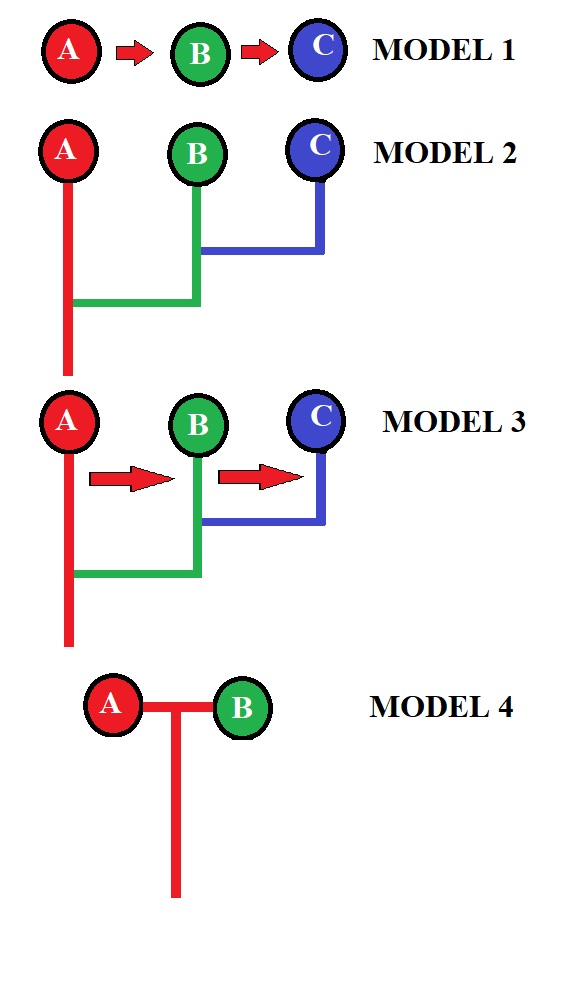

Supplement: Supplementary file 1 [file genes-13-00532-s001.zip › Supplementary Figure S4. Models.jpg]
